# The loss-of-function *GLABROUS 3* mutation in cucumber is due to LTR-retrotransposon insertion in a class IV HD-ZIP transcription factor gene *CsGL3* that is epistatic over *CsGL1*

**DOI:** 10.1186/s12870-015-0693-0

**Published:** 2015-12-29

**Authors:** Yupeng Pan, Kailiang Bo, Zhihui Cheng, Yiqun Weng

**Affiliations:** Horticulture Department, University of Wisconsin, Madison, WI 53706 USA; Horticulture College, Northwest A&F University, Yangling, 712100 China; USDA-ARS, Vegetable Crops Research Unit, 1575 Linden Drive, Madison, WI 53706 USA

**Keywords:** Cucumber, *Cucumis sativus*, Trichome development, Homeodomain leucine zipper protein, HD-ZIP, Map-based cloning, LTR retrotransposon

## Abstract

**Background:**

Trichomes, developed from the protodermal cells (the outermost cell layer of the embryo), are hair-like structures covering the aerial parts of plants. The genetic network regulating trichome development has been extensively studied and well understood in the model species *Arabidopsis thaliana,* which bears unicellular, non-glandular and branched trichomes. However, little is known about the genetic and molecular basis of organogenesis of multi-cellular trichomes in plant species like cucumber (*Cucumis sativus* L.), which are likely different from Arabidopsis.

**Results:**

We identified a new trichome mutant in cucumber which exhibited a completely glabrous phenotype on all aerial organs. Genetic analysis indicated that the glabrous phenotype was inherited as a single recessive gene, *csgl3*. Fine genetic mapping delimited the *csgl3* locus into a 68.4 kb region with 12 predicted genes. Genetic analysis, sequence alignment and allelic variation survey in natural populations identified *Csa6G514870* encoding a class IV homeodomain-associated leucine zipper (HD-ZIP) transcription factor as the only candidate for *CsGL3*, which was 5188 bp in length with 10 predicted exons. Gene expression analysis revealed the loss-of-function of *CsGL3* in the mutant due to the insertion of a 5-kb long terminal repeat (LTR) retrotransposon in the 4th exon of *CsGL3*. Linkage analysis in a segregating population and gene expression analysis of the *CsGL1* and *CsGL3* genes in *csgl1*, *csgl3*, and *csgl1 + 3* genetic backgrounds uncovered interactions between the two genes. Phylogenetic analysis among 28 class IV HD-ZIP protein sequences from five species placed cucumber *CsGL3* into the same clade with 7 other members that play important roles in trichome initiation.

**Conclusions:**

The new glabrous mutation in cucumber was controlled by a single recessive locus *csgl3*, which was phenotypically and genetically distinct from two previously reported glabrous mutants *csgl1* and *csgl2*. The glabrous phenotype in *csgl3* was due to insertion of an autonomous, active, class I transposable element in *CsGL3*, a class IV HD-ZIP transcription factor. *CsGL3* was epistatic to *CsGL1. CsGL3* seemed to play important roles in cucumber trichome initiation whereas *CsGL1* may act downstream in the trichome development pathway(s). Findings from the present study provide new insights into genetic control of trichome development in cucumber.

**Electronic supplementary material:**

The online version of this article (doi:10.1186/s12870-015-0693-0) contains supplementary material, which is available to authorized users.

## Background

Trichomes, developed from the protodermal cells (the outermost cell layer of the embryo), are hair-like structures covering the aerial parts of plants such as leaves, stems, petioles, sepals, petals, ovaries, fruits and seeds. Trichomes are very diverse in shape, size, structure, location, capability to secrete, and functions. Trichomes may play important roles in protecting plants from environmental stresses such as heat, low temperature, high UV, and insect herbivory [1, 2]. Seed trichomes may facilitate seed dispersal. For some specialty crops, such as cucumber (*Cucumis sativus* L.), the presence or absence of trichomes constitutes an important quality issue for the end product.

For convenience, trichomes are often classified as glandular or non-glandular, unicellular or multicellular, and branched or unbranched. The unicellular, non-glandular trichome of *Arabidopsis thaliana* has been used as a model system to study the molecular genetic mechanisms of trichome organogenesis, which involves a transcriptional network consisting of three groups of transcription factors: R2R3 MYBs, the basic helix-loop-helix (bHLH) factors and the WD40 repeat (WDR) proteins (reviewed by [3–5]). Among various components in this network, one class of transcription factors (TFs), the homeodomain leucine-zipper proteins (HD-ZIP), are important players in trichome initiation and development. Based on the domain structures and associated functions, the HD-ZIP proteins are grouped into four classes (I to IV) which share the conserved HD and ZIP domains that are responsible for DNA binding and protein-protein interactions, respectively [6]. Classes III and IV proteins contain two additional domains, a steroidogenic acute regulatory protein-related lipid transfer (START) domain hypothesized to bind sterols and lipids and a SAD (START-associated domain) with unknown functions [6]. The class IV HD-ZIP TFs seem to play critical roles in regulating the differentiation of the epidermis in numerous tissues. The Arabidopsis genome contains 16 class IV HD-ZIP family members including *ARABIDOPSIS THALIANA MERISTEM LAYER1* (*ATML1*)*, GLABRA2* (*GL2*), *ANTHOCYANINLESS2* (*ANL2*)*, PROTODERMAL FACTOR2* (*PDF2*) and *HOMEODOMAIN GLABROUS1* (*HDG1*) through *HDG12*. Most class IV HD-ZIP TFs are expressed specifically in the outer cell layer of the plant organs in which they play a role [7]. *GL2*, *ATML1* and *PDF2* were among the first that have been well characterized. They are thought to be involved in establishing cell fates in the epidermal layer through the regulation of cell layer-specific gene expression. *GL2* seems to be required for trichome differentiation and maintenance, but it is dispensable for trichome initiation [8]. *ATML1* and *PDF2* are a pair of functionally redundant, paralogous genes which are expressed in all cells of the proembryo from the one-cell stage to the 16-cell stage, when their expression becomes progressively restricted to the outer cell layer [9, 10]. *HDG11* and *HDG2* are important for normal trichome development [7]. There is also functional redundancy between *GL2* and *HDG11*, and *GL2* transcript levels are maintained through a positive feedback loop involving *GL2* activation of *MYB23* [11].

The class IV HD-ZIP TF genes have also been identified in several crop species including maize (*Zea mays*) [12], tomato (*Solanum lycopersicum*) [13], cotton (*Gossypium* spp) [14, 15], and rice (*Oryza sativa*) [16]. In cucumber, trichomes cover almost all aerial organs such as the hypocotyl, cotyledons, true leaves, stem, tendrils, flowers, and fruits. Trichomes on the fruits are commonly called fruit spines, and are an important trait in assessment of cucumber fruit quality in different market classes. For example, fruits with large, sparse spines are preferred for American pickles. The north China fresh market type cucumber fruits are covered with dense small spines, and the European greenhouse type or mini (beit alpha) cucumbers are often glossy and smooth with fine, nearly invisible hairs. Despite of its importance in cucumber breeding, little is known about the genetic or regulatory mechanisms of fruit spine or trichome development in cucumber.

Several spontaneous glabrous mutants in cucumber have been reported and characterized. The first one is “*cucumber glabrous-1*(*csgl1*)” or “*micro-trichome* (*mict*)” [17, 18]. The *csgl1* mutant shows no observable trichomes on leaves, stems, tendrils, and floral organs, but has obvious trichomes on the hypocotyl. Under an SEM, many papillae could be observed on the epidermis of the mutant leaves, with the papillae density similar to the trichome density of the wild type suggesting that *CsGL1* may be involved in foliar trichome development but not initiation [18]. Map-based cloning has revealed that *CsGL1* encodes a Class I HD-ZIP TF, and the loss-of-function *csgl1* is due to a 2649-bp genomic DNA deletion spanning the first and second exons of *CsGL1* [17, 18]. The *tiny branched hair* (*tbh*) mutant reported by Chen et al. [19] is probably the same as *csgl1* (*mict*). The *csgl2* mutant from cucumber germplasm line NCG-042 exhibited glabrous stem, petioles, and leaves whereas the surface of the fruits, sepals, fruit peduncles and pedicel of flowers were covered with sparse and fine hairs [20]. More recently, Zhao et al. [21] reported a spontaneous “*trichome-less* (*tril*)” mutant that was completely free from trichomes on all aerial organs, which is true even under an SEM suggesting the *Tril* gene may function in trichome cell fate determination [18, 21, 22].

In Arabidopsis, mature leaf trichomes are characteristically large branched hair cells whose nuclei have undergone multiple rounds of endoreplication and are present on the leaf surface in a nonrandom regular distribution [23]. In contrast, cucumber trichomes are multicellular and non-glandular with malformed organelles and do not undergo endoreplication in development [19]. The role on trichome development by a class I HD-ZIP TF like *CsGL1* in cucumber [17] has not been found in Arabidopsis. These observations suggest that trichome development in cucumber may be regulated by distinct mechanisms from those in Arabidopsis. Here, we reported the identification, map-based cloning and characterization of a new trichome mutant in cucumber, *CUCUMBER GLABROUS 3* (*csgl3*). We presented evidence that the loss-of-function of *CsGL3* was due to the insertion of a 5-kb long tandem repeat (LTR) retrotransposon and *CsGL3* may be involved in determination of the trichome cell fate.

## Results

### The spontaneous mutation in RIL-46 M was controlled by a single recessive gene *csgl3*

In the 2013 winter greenhouse season, one glabrous plant, RIL-46 M (mutant) was found in the recombinant inbred line RIL-46 (F_6_) from WI2757 × True Lemon mating. The self-pollinated progeny of RIL-46 M remained glabrous. The plants at the previous generation (F_5_) of RIL-46 were segregating for this trait at roughly 3 non-glabrous to 1 glabrous (data not shown). F_3_ and F_4_ plants in the pedigree of RIL-46 were all of wild type (non-glabrous, RIL-46 W hereinafter). Glabrous plants were never observed in the two parental lines WI2757 and True Lemon, each of which had been selfed for at five generations. Both RIL-46 M and RIL-46 W were gynoecious. To eliminate the possibility that the glabrous allele was introduced from other pollen sources (which was very unlikely in the greenhouse), we genotyped RIL-46 M and RIL-46 W with 238 highly polymorphic SSRs that were used in polymorphic screening in genetic mapping of this gene (see below), and no polymorphism was found between the two sibling lines. These data supported that RIL-46 M was a spontaneous mutation occurring at the F_3_ generation during the development of WI2757 × True Lemon RILs. This also suggested that RIL-46 M and RIL-46 W were near isogenic lines (NILs) at the glabrous mutation locus.

**Fig. 1:**
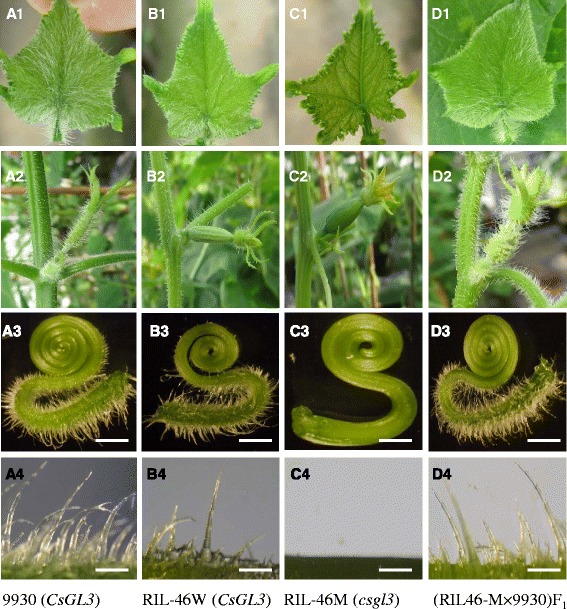
Trichome phenotypic characterization of different cucumber lines. Images of cucumber inbred lines 9930 (A), RIL-46 W (B), RIL-46 M (C) and RIL-46 M × 9930 F_1_ (D). For each line, trichomes of emerging young leaves (A1–D1), female flowers and stem (A2–D2), tendrils (A3–D3) and the hypocotyl (A4–D4) are shown. Bar = 1 mm in A3 to D3; bar = 100 μm in A4 to D4

**Fig. 2:**
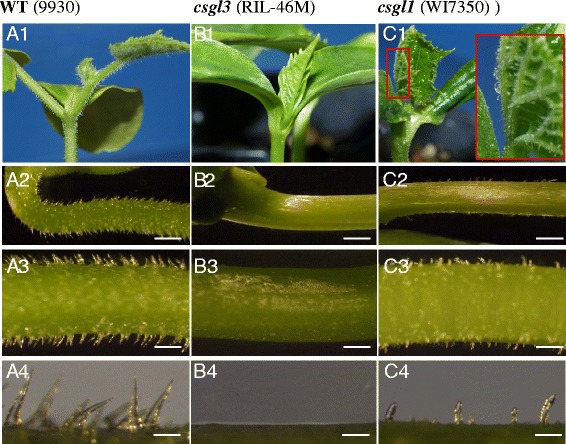
Trichomes on hypocotyls and unexpanded true leaves on 9930 (WT, *left*), RIL-46 M (*csgl3*, *middle*), and WI7350 (*csgl1*, *right*) glabrous mutants. RIL-46 M is completely glabrous on all aerial parts (B1-B4) whereas WI7350 exhibits sparsely distributed trichomes on both hypocotyl and true leaves (C1 to C4). Bar = 1 mm (A2, B2, C2); Bar = 500 μm (A3, B3, C3); Bar = 100 μm (A4, B4, C4)

The trichomes on RIL-46 M, RIL-46 W, 9930 as well as RIL-46 M × 9930 F_1_ were examined visually or with a dissecting or electron microscope. Representative images of the true leaves, tendrils, stems, ovaries of these materials are shown in Fig. 1. As compared with the wild type 9930 and RIL-46 W (Fig. 1A and B), the hypocotyl, cotyledons, true leaves and petioles, the stem, tendrils, sepals and pedicles of flowers, ovaries, fruits, and fruit peduncles of RIL-46 M mutant plants were all free from trichomes (Fig. 1C and Fig. 2A and B). On the other hand, except for the glabrous phenotype in RIL-46 M, there were no observable differences between the two NILs in growth habit, growth vigor or growth rate, flowering time, fruit and seed setting indicting no obvious pleiotropic effects of this trichome mutation on other traits.

**Fig. 3:**
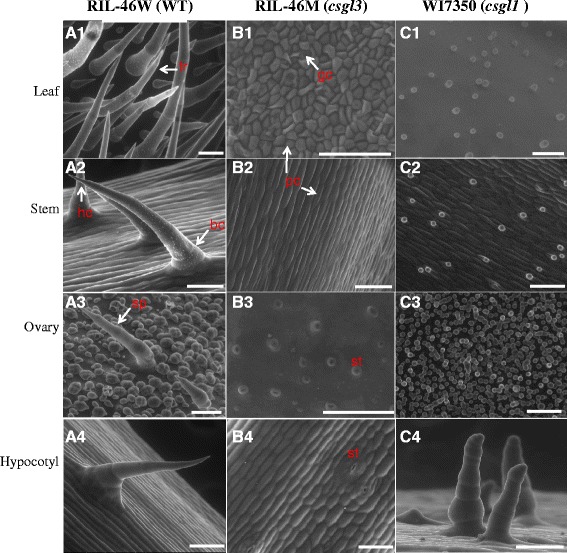
ESEM images of trichomes on young leaf, stem, ovary and hypocotyl of RIL-46 W (WT, A1–A4), RIL-46 M (*csgl3* mutant, B1–B4) and WI7350 (*csgl1* mutant, C1–C4). As compared with WT, *csgl1* mutant has aberrant trichome cells that fail to develop into mature trichomes whereas *csgl3* mutant completely lacks trichome cell development. The trichomes on hypocotyl of the *csgl1* mutant were relatively well developed but the head and base cells of each trichome (C4) were morphologically different from those in WT (A4). Sp = fruit spine, bc = base cell, hc = head (apical) cell, tr = trichomes, gc = guard cells, pc = pavement cells, st = stomata. Bars = 100 μm

A noticeable difference between RIL-46 M (*csgl3*) and the *csgl1* glabrous mutant WI7350 was the distribution of trichomes on the hypocotyl and emerging true leaves (especially leaf veins) in WI7350 (Fig. 2B and C). Under a dissecting microscope, while RIL-46 M was trichome free, WI7350 exhibited short and sparsely distributed trichomes, although less pronounced than the WT (Fig. 2a). Consistent with these observations, under an ESEM (Fig. 3), while the WT RIL-46 W exhibited many typical multicellular trichomes on the epidermis of all organs examined (leaf, stem, ovary and hypocotyl, Fig. 3a1–a4), only tiny trichomes with aberrant cells were seen in *csgl1* mutant (Fig. 3c1–c4), and no trichomes could be observed in the *csgl3* mutant (Fig. 3B1-B4). In addition, the trichomes on the hypocotyl in the *csgl1* mutant were obvious except for the head (apical) cell and base cells of each trichome that did not seem well developed. These results clearly suggested that RIL-46 M is a distinct mutant from *csgl1*.

Segregation data in four F_2_ or BC_1_ populations from different crosses are presented in Table 1. The F_1_ plants from both 9930 × RIL-46 M (Fig. 1d) and Gy14 × RIL-46 M showed no differences in trichome morphology and density as compared with its wild type parental lines (Gy14 and 9930) (Fig. 1a) indicating the recessive nature of the mutation in RIL-46 M. Among 665 F_2_ plants from the 9930 × RIL-46 M cross, 484 and 181 showed wild type and completely glabrous phenotype, respectively. This was consistent with the expected 3:1 segregation (*P* = 0.1865 in χ^2^ test). Similarly, the segregation in the BC_1_P_1_, BC_1_P_2_, and Gy14 × RIL-46 M F_2_ populations all agreed with a single recessive gene underlying the glabrous phenotype in RIL-46 M (Table 1). In light of the phenotypic differences of this mutant with previously reported *csgl1* (*mict*) and *csgl2*, this new mutation was designated as *csgl3*.

**Table 1:** Phenotypic segregation at the *CsGL3* locus among different populations

Populations	# Plants examined	Wild type (*CsGL3*_)	Glabrous (*csgl3csgl3*)	Expected ratio (WT: mutant)	*P*-value
(9930 x RIL-46 M) F_2_	665	484	181	3:1	0.1865
BC1P1 (9930× RIL-46 M)F1 × RIL-46 M	341	177	162	1:1	0.4125
BC1P2 (9930× RIL-46 M)F1 × 9930	96	96	0	1:0	-
(Gy14 × RIL-46 M) F_2_	384	283	101	3:1	0.5557

### Fine mapping identified a class IV HD-ZIP TF as the candidate gene for *csgl3*

**Fig. 4:**
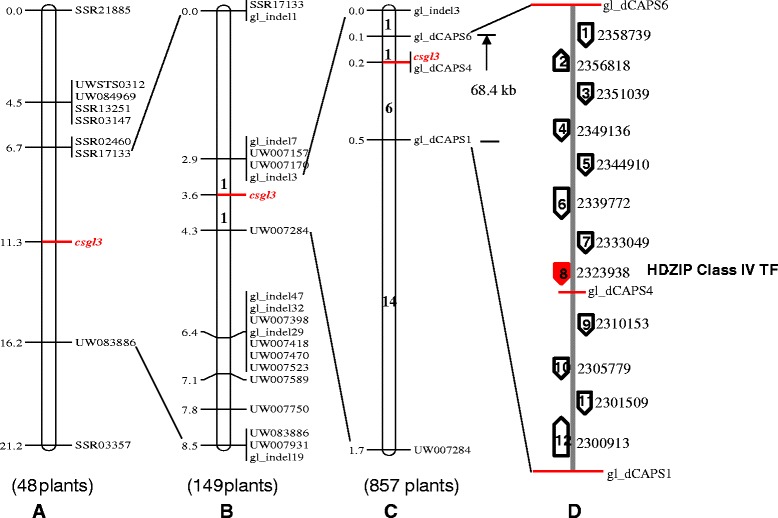
Fine genetic mapping of the *csgl3* locus. A 68.4 kb region in chromosome 6 was identified to harbor the *csgl3* gene with genetic mapping by stepwise increase of the population size and scaffold-based chromosome walking (**a**, **b** and **c**). Twelve genes were predicted in the 68.4 kb region and the 8th, a class IV HD-ZIP TF was the candidate gene for *CsGL3* (**d**). Numbers to the left of the chromosome are genetic distance in cM. Numbers within the chromosome bars in B and C are number of recombinants in the interval

From 46 BC_1_P_1_ plants of RIL-46 M × 9930 (Table 1), two DNA pools, the M-pool (glabrous) and the WT-pool (non-glabrous), were constructed. Among 238 SSR markers tested, 6 were polymorphic between the two pools: SSR03918 and SSR13466 were located on chromosome 3 and the other four (SSR21885, SSR03147, SSR13251 and SSR02460) on chromosome 6. The two chromosome-3 markers were excluded after linkage analysis with 48 BC_1_P_1_ plants (data not shown). Thus, initial mapping placed the *csgl3* locus in chromosome 6 linked with four markers with SSR02460 being the closest. Since SSR02460 was physically located in the Gy14 scaffold00542 and 9930 scaffold000002, 53 additional SSR markers from the two scaffolds were tested, and three (SSR17133, UW083886 and SSR03357) were polymorphic between the two pools. Linkage analysis of the 7 markers in 46 BC_1_P_1_ plants identified SSR17133 and UW083886 flanking the *csgl3* locus at a distance of 4.6 and 4.9 cM, respectively (Fig. 4a). The physical distance between the two flanking markers was 1.9 Mbp in 9930 scaffold000002. Information about these and all other markers used in the present study is provided in Additional file 1: Table S1.

The RIL-46 M mutant was derived from the cross of WI2757 with True Lemon. To identify the origin of the nearly 1.9 Mbp DNA fragment harboring the *csgl3* locus, we re-sequenced the genomes of both parental lines. The Illumina short sequence reads were aligned with Gy14 scaffold00542 reference; 11 indel and 24 SSR markers [24] within the 1.9 Mbp region were identified, which were polymorphic between WI2757 and True Lemon and used to genotype RIL-46 M. All 35 markers were polymorphic between RIL-46 M and True Lemon, but monomorphic between RIL-46 M and WI2757 suggesting this 1.9 Mbp region was originated from WI2757. Therefore, the WI2757 resequencing reads were employed in subsequent marker development for fine mapping of *csgl3*. From 138 SSRs and 63 indels in this region, 17 new polymorphic markers were identified. A linkage map (Fig. 4b) was developed with these markers in 149 BC_1_P_1_ plants. Now the *csgl3* locus was flanked by gl_indel3 and UW007284 which were 210 kb apart in 9930 scaffold000002.

A new set of 665 F_2_ plants was screened with gl_indel3 and UW007284, and 22 recombinants were identified in this interval (Fig. 4c). Six SNPs between WI2757 and 9930 in the 210 kb region were employed to develop dCAPS markers, of which three (gl_dCAPS1, gl_dCAPS4 and gl_dCAPS6) were successfully mapped. Linkage analysis revealed that gl_dCAPS4 was co-segregating with *csgl3*, whereas gl_dCAPS1 and gl_dCAPS6 flanked the *csgl3* locus at 0.1 and 0.3 cM, respectively, which was approximately 68.4 kb physically.

We annotated this 68.4 kb genomic DNA region and 12 genes were predicted (Fig. 4d). Information about the position and predicted functions of each gene is presented in Additional file 1: Table S2. To pinpoint possible candidate gene(s) of *csgl3*, we first looked into sequence variations in this 68.4 kb region by alignment of the genomic DNA sequence of WI2757 to 9930 scaffold000002. Ten SNPs or indels were identified, of which 9 were located in the intergenic region and one in the first intron of the 11th predicted gene suggesting that these sequence variations are unlikely associated with the glabrous mutation in RIL-46 M. We further conducted sequence alignment of this 68.4 kb region with 10 other re-sequenced, non-glabrous cucumber lines. No consistent marker-phenotype association was found among these lines (data not shown), which provided additional evidence that the 10 SNPs or indels were not associated with the *csgl3* mutation.

**Fig. 5:**
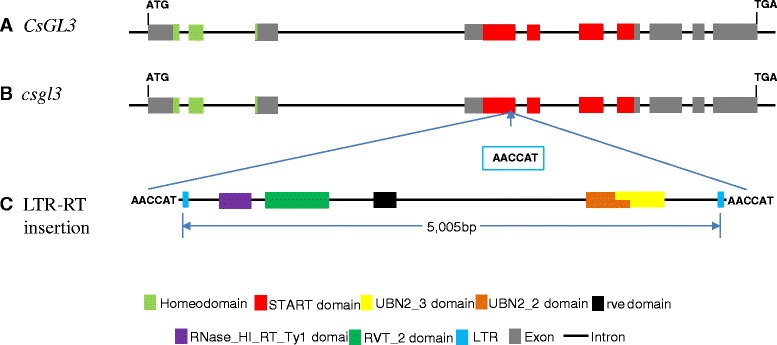
Predicted gene structure of the wild type (**a**) and mutant (**b**) alleles of *CsGL3* candidate gene (HD-ZIP Class IV TF) and annotated 5005-bp LTR retrotransposon (**c**). Boxes and lines indicate exons and introns, respectively. There are 10 exons in the predicted gene, and the mutant allele is due to insertion of 5005-bp LTR-RT at the 4th exon (**b**). The LTR-RT is predicted to encode all protein domains required for active transposition (**c**). Boxes or lines are not drawn to scale

**Fig. 6:**
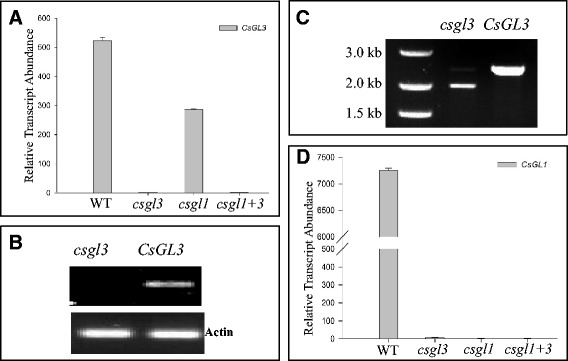
Relative transcript abundances of *CsGL1* and *CsGL3* genes in different genetic backgrounds (WT, *csgl3*, *csgl1* and *csgl1 + 3*). **a** Expression of *CsGL3* is nearly undetectable in *csgl3* (**a**, **b**) and *csgl1 + 3* double mutant (**a**), and it is reduced by approximately half in *csgl1* mutant. **b** Semi RT-PCR of *CsGL3* between RIL-46 M and RIL-46 W suggests the transcript of 4th exon is missing in the mutant. **c** PCR cloning of the full-length cDNA of *CsGL3* between RIL-46 M and RIL-46 W suggests missing of the 4th exon sequence in the transcript of the mutant line. **d** Expression of *CsGL1* was almost undetectable in all mutant lines

Among the 12 annotated genes in the 68.4 kb region, the 8th one (*Csa6G514870*) was predicted to encode a member of the class IV HD-ZIP TF. In the 9930 draft genome, this gene was 5188 bp in length with 10 exons (Fig. 5a) and encoded a protein of 721 amino acids with the conserved homeodomain (amino acids 49–108) and START domain (amino acids 232–458). We investigated its expression in the apical buds of RIL-46 W (*CsGL3*) and RIL-46 M (*csgl3*) with qPCR (Fig. 6a). The expression level of the *CsGL3* candidate gene was nearly 500 times as high in the WT as in the *csgl3* mutant where it was almost undetectable. Consistent with this, when we ran a semi quantitative RT-PCR analysis of the *CsGL3* candidate gene using primer pair GL3_RT2 spanning the 4th and 5th exons (Table S1), the PCR product in RIL-46 M was not detectable in agarose gel electrophoresis whereas the band of RIL-46 W was bright and strong (Fig. 6b) suggesting that the glabrous mutation in RIL-46 M was probably due to change(s) in exons 4 and 5 of the *CsGL3* candidate gene resulting in the loss-of-function of this gene.

We cloned cDNA sequences of *CsGL3* from RIL-46 W and RIL-46 M (primers GL3_CDS, Table S1). The PCR product from RIL-46 W had the expected full length, but the band from RIL-46 M mutant was ~400 bp shorter (Fig. 6c). Alignment of the cDNA sequences between RIL-46 W and RIL-46 M revealed that the predicted 438-bp 4th exon in *CsGL3* was missing in the *csgl3* mutant.

### The loss-of-function mutation of *CsGL3* is due to insertion of a 5-kb LTR retrotransposon

We cloned the *CsGL3* genomic DNA sequences from RIL-46 M and RIL-46 W. While the PCR product size in RIL-46 W was expected, primers designed in the 4th and 5th exonic region amplified a DNA fragment that was 5 kb longer in RIL-46 M than in RIL-46 W indicating a large DNA insertion in the mutant. Indeed, sequencing of the full length of *csgl3* allele revealed a 5005 bp insertion in the 4th exon of *CsGL3* resulting in a 10,199-bp fragment in RIL-46 M (Fig. 5b). The complete sequences of *CsGL3* (5188 bp) and *csgl3* (10,199 bp) alleles were provided in Additional file 2. Comparison of the *CsGL3* sequences among RIL-46 M, RIL-46 W and WI2757 revealed no sequence variations except for the 5005 bp insertion in RIL-46 M.

In the *csgl3* genomic DNA sequence, the 5005 bp insertion was flanked with the 5′-AACCAT-3′ insert Target Site Duplication (TSD). Self-alignment of this sequence with dot-plot revealed the presence of ~200 bp long terminal repeats (LTRs). Indeed, alignment between the first and last 300 bp of the insertion confirmed the presence of a 222-bp LTR beginning with a 5′-GT-3′ and ending with a 5′-TA-3′. The LTRs shared 100 % sequence identity with one another. Annotation of this 5005 bp sequence suggested that this LTR retrotransposon (LTR-RT) had a complete gene structure with five exons and four introns, and the coding regions were predicted to encode four conserved protein domains including RNase_HI_RT_Ty1, RVT_2, rve and UBN2 (Fig. 5c), which are typical of LTR-RTs [25]. According to the classification of transposable elements (TE) in plant genomes [26], this LTR-RT was an autonomous, class I/*Copia* type TE which seemed to be active in the RIL-46 M genome.

### The 5-kb LTR-RT was copious in the cucumber genome but the insertion at the *CsGL3* locus in RIL-46 M was unique in natural populations

**Fig. 7:**
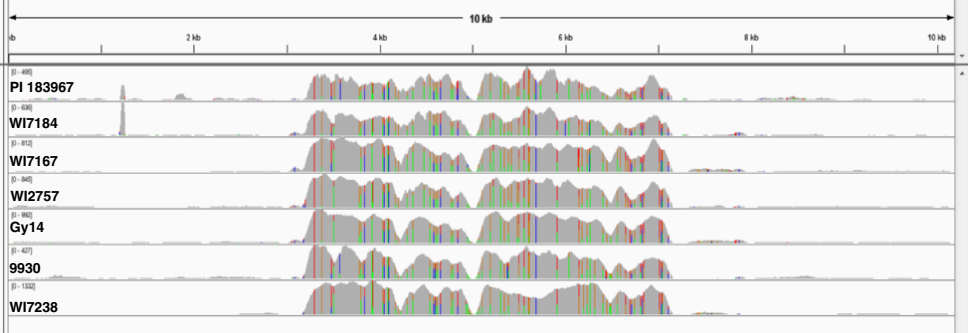
Distribution of the 5005-bp LTR-RT in the cucumber genome. Alignment of Illumina short reads of seven cucumber lines against the *csgl3* mutant allele indicates multiple copies of this LTR-RT in the se-sequenced genoems

LTR-RTs are widely present in plant genomes and play important roles in genome evolution [27]. To assess the distribution of this LTR-RT in the cucumber genome, using the 10,199 bp *csgl3* sequence as the reference, we aligned the Illumina short reads of 7 cucumber lines of different botanical varieties including a wild (var *hardwickii*, PI 193967), two semi-wild Xishuangbanna (var. *xishuangbannesis*, WI7167 and WI7184) and four cultivated (var. *sativus*, Gy14, 9930, WI2757 and WI7238) cucumber lines. The frequency distribution of raw reads in each re-sequenced genome (Fig. 7) suggested that this LTR-RT is presented in each genome in much higher copies than surrounding sequences. In addition, no significant variations of copy numbers were observed in different botanical varieties indicating this LTR-RT existed well before the divergence of different cucumber linages. We also BLASTed this LTR-RT sequence in the draft genome assemblies of Gy14 and 9930 cucumbers, as well as melon (*C. melo* L.), and found multiple copies of this sequence in all genome assemblies although it was difficult to determine if the complete whole 5005-bp sequence was present in the assemblies (data not shown).

To confirm the identity of the 5 kb LTR-RT insertion with the glabrous mutation in RIL-46 M, we investigated the allelic diversity at *csgl3* locus in natural populations. Three primers were designed including two (GL3_2L, and GL3_2R) from the 4th and 5th exons of the *CsGL3* gene flanking the insertion point, and one (INSERT_R) within the LTR-RT (see Table S1 for primer sequence information). Duplex PCR with the three primers allowed identification of wild type (no LTR-RT insertion, expected size 461 bp) and mutant type homozygotes (with insertion, expected size 917 bp), as well as heterozygotes (both bands) at the *CsGL3* locus. Among 384 cucumber lines examined, all amplified the 461 bp fragment (see Additional file 3: Figure S1 for representative gel profiles), which was consistent with the non-glabrous phenotype of these lines. This provided additional evidence that the 5005 bp insertion in *CsGL3* was indeed the casual mutation for the glabrous phenotype in RIL-46 M.

### *CsGL3* is epistatic to *CsGL1* in trichrome organogenesis

We developed a segregating population from the cross between two glabrous mutants RIL-46 M (*csgl3*) and WI7350 (*csgl1*). The F_1_ was wild type (non-glabrous). In F_2_ plants, three phenotypes could be recognized: WT, *csgl1*-type that had the characteristic trichomes on the hypocotyl and petioles of unexpanded leaves, and *csgl3*-type which was completely free from trichomes on any aerial organs (Figs. 1, 2 and 3). Among 89 F_2_ plants, 50, 13 and 26 were WT, *csgl1*-type and *csgl3*-type, respectively, which was consistent with a segregation ratio of 9:3:4 (χ^2^ = 0.7495, *P* = 0.6875). These results indicated that *csgl1* and *csgl3* were two independent, recessively inherited loci, and *csgl3* seemed to be epistatic to *csgl1* in phenotypic expression.

We investigated the expression of both genes in the *csgl1*, *csgl3* and *csgl1 + 3* genetic backgrounds. The double mutant *csgl1 + 3* carrying both genes were identified from the above-mentioned F_2_ population with gene-specific molecular markers (see Table S1 for primer sequence information). The expression levels of *CsGL3* and *CsGL1* in three genetic backgrounds were illustrated in Fig. 6a (for *CsGL3*) and Fig. 6d (for *CsGL1*). Both *CsGL1* and *CsGL3* were highly expressed in the apical buds in WT, and almost undetectable in the *csgl1 + 3* (double mutant) background. *CsGL1* had practically no expression in either *csgl1* or *csgl3* background. *CsGL3* showed minimal expression in the *csgl3* background, and its expression was reduced by nearly half in the *csgl1* background as compared with that in WT (Fig. 6a). From these results, it was evident that *CsGL1* and *CsGL3* had interactions with each other: while the expression of *csgl1* was dependent on *csgl3* genetic background, the expression of *csgl3* was also affected by *csgl1*, which was consistent with the epistatic effect of *csgl3* over *csgl1* revealed from the segregating data.

### Phylogenetic analysis grouped cucumber CsGL3 with class IV HD-ZIP homologs in other species with similar functions

**Fig. 8:**
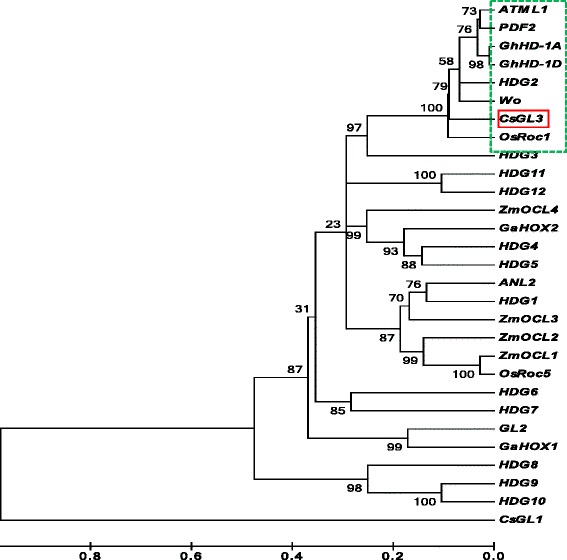
Phylogram of cucumber CsGL3 and 27 other class IV HD-ZIP TF proteins. Among the 27 proteins, 16 are from Arabidopsis (ATML1, PDF2, ANL2, GL2, and HDG1- HDG12); 2 from rice (OsRoc1 and 5), 4 from cotton (GaHOX1, GaHOX2, GhHD-1A, and GhHD-1D), 4 from maize (ZmOCL1 to 4), and 1 from tomato (Wo). The cucumber class I HD-ZIP protein CsGL1 is used as an outlier in phylogenetic analysis. The Neighbor-joining tree is constructed with the MEGA 5.0 software (http://www.megasoftware.net/) with 1000 bootstrap replications. The numbers at each node is the probability that this node is supported (in percentages)

To understand the structural and functional relationships between CsGL3 in cucumber and class IV HD-ZIP proteins in other species, we conducted phylogenic analysis of CsGL3 with 27 other HD-ZIP class IV TFs including 16 from Arabidopsis (ATML-1, PDF2, ANL2, GL2, HDG1 to HDG12), 1 from tomato (WO), 4 from maize (ZmOCL1 to ZmOCL4), 2 from rice (OsRoc1, OsRoc5), and 4 from cotton (GhHD-1A, GhHD-1D, GaHOX1, and GaHOX2). The cucumber CsGL1 was used as an outlier. The neighbor-joining tree is shown in Fig. 8. It was clear that clustering of these sequences was based first on their functions and then on their phylogenetic distances. ATML-1 and PDF2 are two paralogs arisen from Arabidopsis genome duplication that are indispensable for epidermal cell-fate specification in the embryos [10, 28]. Cucumber CsGL3, tomato WO and cotton GhHD1A, and GhHD-1D were in the same clad as ATML-1 and PDF2, which all play important roles in trichome (or cotton fiber) initiation (see discussion below). CsGL3 had 41.6, 70.7, and 66.5 % amino acid sequence identity with GL2, PDF2, and WO, respectively. Interestingly, OsRoc1 from rice, a monocot, was grouped in the same clade as other PDF2-like proteins from dicot species suggesting these proteins were highly conserved in both function and structure in flowering plants. This also implied that OsRoc1 may play a similar role of trichome initiation. On the other hand, the Arabidopsis GL2 that acts downstream of ATML1/PDF2 in the pathways regulating trichome development was much diverged and phylogenetically far away from CsGL3. The diploid cotton GaHOX1 gene, which regualts the fibre development in cotton [14], was the closest one with GL2. The function- and structure-based clustering was also evidenced from the fact that CsGL1, a member of the class I HD-ZIP TF family was separated far away from all class IV HD-ZIP IV members in the phylogenetic tree (Fig. 8).

## Discussion

### Genetic control of glabrous phenotypes in cucumber

In this study, we identified a new glabrous mutant that was controlled by a single recessive gene *csgl3*. Two cucumber glabrous mutants, *csgl1* (*mict*) and *csgl2* have been genetically characterized previously [17, 18, 20]. The three mutants were phenotypically different. While *csgl3* was completely free of trichomes on any aerial organs, the *csgl1* mutant showed trichomes on the hypocotyls and emerging leaves, as well as tiny trichome cells (under ESEM) (Figs. 1, 2 and 3). For the *csgl2* mutant, there were trichomes on the fruits, flower sepals, and fruit peduncle whereas the stem, leaf and petiole were largely glabrous. Chen et al. [19] described a spontaneous mutant, *tiny branched hair* (*tbh*) which had tiny and branched trichomes with increased density and aberrant cell shape. Zhao et al. [21] identified another mutant, *trichome-less* (*tril*) that was completely free of hairs. Based on the descriptions, the *tbh* and *tril* mutants probably corresponded to the *csgl1* and *csgl3* mutants, respectively. Clearly the three glabrous mutants were under the control of different genetic mechanisms. Indeed, *csgl1*, *csgl2* and *csgl3* were located in chromosomes 3, 2, and 6, respectively. While the nature of *CsGL2* is unknown, *CsGL1* and *CsGL3* have been shown, respectively, to encode a class I and IV HD-ZIP transcription factor ([17, 18] and this study).

Arabidopsis trichomes are unicellular but undergo a complex developmental process including four rounds of endoduplication resulting in a genomic DNA content of 32C in trichome cells [3, 23]. In contrast, cucumber trichomes are multicellular which do not undergo any endoduplication in morphogenesis [19]. The role of class I HD-ZIP TF gene *CsGL1* in trichome development in cucumber has not been reported in Arabidopsis [17]. Differentially expressed genes identified in transcriptome profiling in the *tbh* mutant and its wild type were also different from what was observed in Arabidopsis [19]. These results suggest that there may be different mechanisms regulating trichome organogenesis between cucumber and Arabidopsis [17, 19]. Therefore, the *csgl3* mutant reported herein may provide a valuable tool in understanding the regulatory network for trichome development in cucumber.

### Mutagenesis induced by LTR-RT insertion in *CsGL3* of cucumber

Through map-based cloning, we identified a class IV HD-ZIP TF as the candidate gene for *CsGL3*. We showed that the loss-of-function of *CsGL3* was due to the insertion of a 5005-bp LTR-RT (Fig. 5). LTR-RTs are structurally similar to retroviruses that can move within a host genome through an RNA intermediate by a “copy-and paste” mechanism [26, 27]. At a single gene level, TE insertions can result in loss of gene function, reprogramming of gene expression, gene deletions, rearrangements or transposition, coding-sequence exaptation and epigenetic effects, all of which have been well documented in a number of plant species (reviewed in [28]). In cucumber, there was one report on TE-induced mutagenesis in which the powdery mildew resistance was the result of the insertion of a 1449-bp LTR-RT in the 11th exon of the *MLO-like* gene major-effect QTL, *pm5.1* in chromosome 5 [29].

Most TE insertion-induced mutations detected were historical events, but in the present study, examination of the pedigrees suggested that the LTR-RT insertion in RIL-46 M occur in the F_3_ generation during RIL development from the cross of WI 2757 with True Lemon. Thus this was the first example that the action of a LTR-RT insertion causing loss of function of a gene was caught “red handed”. Consistent with this, sequence analysis of this 5005-bp insertion revealed the presence of 100 % identical LTRs and TSDs. The fact that the LTRs are completely identical to one another is an indication that the TE is relatively recently inserted. This LTR-RT was predicted to encode all necessary proteins for transposition (Fig. 5c) suggesting it is an autonomous and active retroelement in RIL-46 M.

This autonomous, active LTR-RT seemed to be unique to the RIL-46 M lineage, but non-autonomous and inactive in other cucumber lines. Sequence alignment indicated that this LTR-RT existed in multiple copies in seven re-sequenced cucumber genomes (Fig. 7) as well as in the Gy14 and 9930 cucumber and melon [30] draft genomes. These observations suggested that the LTR-RT sequence is widely distributed in the genomes of the *Cucumis* species, which were diverged from a common ancestor ~12 million years ago [31]. No intact 5005-bp full length sequence was found in the Gy14, 9930 or melon draft genomes, though. Using the predicted 2994-bp coding sequence (CDS) in this LTR-RT as query, we BLASTed the Gy14 cucumber leaf and root transcriptomes containing more than 2.3 million ESTs from Roche/454 sequencing; no EST hits covered the full length CDS (data not shown) indicating this LTR-RT may not be active in the Gy14 genome.

### Possible roles of *CsGL3* in cucumber trichome initiation and development

The CsGL3 protein shared 70.7 % amino acid sequence identity with that of Arabidopsis PDF2, both of which were members of the class IV HD-ZIP TF family. Phylogenic analysis placed CsGL3 in the same clad as PDF2/ATML1, HDG2 of Arabidopsis, GhHD-1A and GhHD1D of cotton, and OsROC1 of rice (Fig. 8). In contrast, the Arabidopsis GL2, which played a pivotal role in trichome morphogenesis after trichome initiation [32, 33] showed lower sequence identity (41.6 %) with CsGL3 and sat at a distant location in the phylogenetic tree (Fig. 8).

The Arabidopsis *PDF2* is expressed exclusively in the L1 cells (layer 1, the outermost cell layer) of the shoot apical meristem (SAM). *PDF2* and *ATML1* are functionally interchangeable and work together to regulate the molecular pathway required for the differentiation of the protodermal cell layer in the embryo [10, 28, 34, 35]. Several other class IV HD-ZIP TF protein that were clustered in the same clad as ATML1 and PDF2 (Fig. 8) probably share the similar functions. For example, the cotton GhHD1-1A and GhHD1-1D, the tomato WO are all involved in trichome (fiber) initiation [14, 15]. Therefore, *CsGL3* may play similar functions in cucumber in cell fate determination and trichome initiation in cucumber.

We showed that *CsGL1* and *CsGL3* were two independent loci with *CsGL3* being epistatic to *CsGL1* (Table 1, Fig. 6). While the expression of *CsGL1* and *CsGL3* were barely detectable in their respective mutant or double mutant (*csgl1 + 3*) backgrounds, the expression of *CsGL3* was significantly decreased in *csgl1* mutant, and *CsGL1* was not detectable in *csgl3* mutant (Fig. 6). *CsGL1* encodes a class I HD-ZIP TF [17, 18]. Zhao et al. [18] hypothesized that *CsGL1*, which shares 52 % sequence identity to *Arabidopsis ATMI1*, may function negatively in trichome spacing and positively in regulating apical cell and stalk cell morphogenesis during trichome development. Thus, *CsGL1* acts downstream of *CsGL3* in the trichome morphogenesis pathway(s), which seem to be consistent with phenotypic variations observed in the two mutants (Figs. 1, 2 and 3). Zhao et al. [21, 22] conducted comparative transcriptome profiling at both *csgl1* (*mict*) and *trichome-less* (*tril*) mutants. When compared with the wild type, 43 and 62 transcription factor genes exhibited significant differential expression in *csgl1* and *tril* mutant, respectively. Among them, the Arabidopsis *GL2-like* and *ATHB-51-like* (Class I HD-ZIP member) genes were down-regulated in both mutants (both were undetectable in the *tril* mutant), whereas the expression of *ATHB-21-like* (Class I HD-ZIP family member) was up-regulated in *csgl1* mutant but not detectable in the *tril* mutant. In Arabidopsis, *GL2* is required for trichome differentiation and maintenance but is dispensable for trichome initiation; the two class I HD-ZIP family member genes *ATHB21* and *ATHB51* are broadly expressed in roots, leaves, stems, flowers, and siliques of adult plants [36]. In cucumber, *GLABRA2*-*like* and *ATHB51*-*like* genes were specifically expressed in the epidermis and trichomes, respectively [21]. These data provided further evidence to support the notion that *CsGL1* and *CsGL3* may play important roles in cucumber trichome development and initiation, respectively.

## Conclusions

A new glabrous mutant, *csgl3*, in cucumber was identified, which was phenotypically and genetically distinct from two previously reported glabrous mutants *csgl1* and *csgl2*. Fine genetic mapping delimited the *csgl3* locus into a 68.4 kb genomic region with a class IV HD-ZIP TF in this region as the best candidate for *CsGL3*. The loss-of-function of *CsGL3* in the mutation was due to the insertion of a 5005-bp autonomous, active LTR-RT in the 4th exon of *CsGL3. CsGL3* was epistatic to the class I HD-ZIP TF gene *CsGL1*, which was responsible for the glabrous mutation *csgl1. CsGL3* seemed to play important roles in trichome initiation whereas *CsGL1* may act downstream in the trichome development pathway(s).

## Methods

### Plant materials and phenotypic data collection

The cucumber inbred line RIL-46 M (a.k.a. WI7412 with *csgl3*) was a spontaneous glabrous mutant found in the F_6_ RIL-46 W (a.k.a. WI7225B-46, wild type, *CsGL3*) from the cross between WI2757 and True Lemon cucumber inbred lines. Marker analysis indicated that RIL-46 W and RIL-46 M were NILs for the *csgl3* locus. For study of the inheritance mode and genetic mapping of the mutation, three segregating populations were developed from the cross of RIL-46 M with a north China type cucumber inbred line 9930 (non-glabrous WT). The F_1_ of 9930 × RIL-46 M was self-pollinated to produce F_2_ or backcrossed with either parent to produce BC_1_P_1_ (F_1_ × RIL-46 M) and BC_1_P_2_ (F_1_ × 9930). To investigate possible effects of genetic backgrounds on expression of *csgl3*, RIL-46 M was crossed with a North American pickling cucumber line Gy14 to generate an F_2_ population. The draft genome assemblies for both Gy14 and 9930 are available [37, 38].

The cucumber glabrous mutant WI7350 carries the *csgl1* gene. To understand the allelic relationships of *csgl1* and *csgl3*, WI7350 was crossed with RIL-46 M to generate an F_2_ population from which the double mutant *csgl1 + 3* carrying both *csgl1* and *csgl3* genes were identified. Seeds of WI7350 were kindly provided by Dr. Junsong Pan, Shanghai Jiaotong University, China.

All plant materials for genetic mapping were grown in the Walnut Street Greenhouse (WSGH) of the University of Wisconsin-Madison under natural photoperiodic condition. The trichome phenotype was evaluated starting from two-week old seedlings throughout the whole growing stage. For trichome phenotype data in BC_1_ and F_2_ plants, χ^2^-tests for goodness-of-fit were used to test for deviations of the observed data from the theoretically expected segregation (1:1 for BC_1_P_1_ and 3:1 for F_2_ populations).

To investigate allelic diversity at the *csgl3* locus in natural populations, the trichome phenotypes of 384 cucumber lines were examined. All lines were grown in the University of Wisconsin Hancock Research Station in Hancock, Wisconsin in 2014 and 2015 summer seasons. At least three plants were surveyed for trichome density and distribution on both vegetative and reproductive organs.

The epidermal structures for various organs (hypocotyl, cotyledons, true leaves, ovaries etc.) of seedling or adult plants were examined with either a dissecting microscope or an ESEM (Model: FEI Quanta) at the University of Wisconsin Newcomb Imaging Center.

### Map-based cloning of *csgl3* in cucumber

For quick identification of molecular markers linked with the *csgl3* locus, BSA was employed in the BC_1_P_1_ population of 9930 × RIL-46 M cross (F_1_ × RIL-46 M) (Table 1). Two DNA pools were constructed: the M-Pool consisting of 12 glabrous plants and the WT-Pool with 12 non-glabrous plants. Two hundred and thirty eight SSR markers evenly distributed in 7 cucumber chromosomes [39] were selected to screen for polymorphisms between the two pools. After initial anchor of *csgl3* in chromosome 6, a scaffold-based chromosome walking strategy was taken to identify more closely linked markers. Draft genome scaffold assemblies from both the 9930 (V2.0) [37] and Gy14 (V1.0) [38] cucumber lines were employed. In the target scaffolds, new markers were selected from the published cucumber genetic maps [40, 41] and a collection of 83,689 SSR markers that were developed from the Gy14 draft genome assembly [39, 42].

At fine mapping stage, SNPs and Indels were explored in the next-generation whole genome re-sequencing data. The genomes of WI2757, True Lemon and 9930 cucumber inbred lines were re-sequenced with the Illumina Hi-Seq 2000 platform at > 15× coverage each (100 bp paired end). For marker discovery, short Illumina sequencing reads were aligned to the 9930 draft genome with the BWA (Burrows-Wheeler Alignment Tool) software package [43]. Indel identification and SNP-calling were performed by SAM tools software [43]. For Indels, only those with ≥ 3 bp differences were utilized for primer design with Primer3web (http://primer3.ut.ee/). For SNP genotyping, dCAPS [44] markers were developed with dCAPS Finder 2.0 [45].

All newly developed markers were first screened for polymorphism with M-pool and WT-pool; polymorphic markers were applied to 48 BC_1_P_1_ plants for linkage analysis, which were then extended to the larger BC_1_P_1_ population (*n* = 149) to identify recombinant plants in the region defined by flanking markers. Very closely linked or co-segregating markers in BC_1_P_1_ were further applied to 665 F_2_ plants.

DNA extraction, PCR amplification of molecular markers and gel electrophoresis was conducted as described in Li et al. [46]. Linkage analysis of the *csgl3* locus with molecular markers was performed with the Kosambi mapping function using JoinMap 4.0 with the threshold LOD score of 4.0.

### DNA sequencing, gene annotation/function prediction, and candidate gene identification

Fine genetic mapping delimited the *csgl3* locus in a 68.4 kb region which was annotated with the computer program FGENESH (http://linux1.softberry.com/berry.phtml/). Function prediction of annotated genes was conducted with BLASTx at the NCBI website (http://blast.ncbi.nlm.nih.gov). DNA sequences between WI2757 and 9930 in this 68.4 kb were aligned to confirm the candidate gene for the *csgl3* locus. Genome DNA and cDNA sequences of the *csgl3* candidate genes, including a 5005-bp retrotransposon insertion were cloned from RIL-46 W and RIL-46 M with the Sanger sequencing method.

### Quantitative reverse transcription PCR (qRT-PCR) analysis

The apical buds from RIL-46 M (*csgl3*), RIL-46 W (WT, *CsGL3*), WI7350 (*csgl1*) and double mutant (*csgl1 + 3*) were collected and flash frozen in liquid nitrogen. Total RNA was isolated with RNeasy Plant Mini Kit (Qiagen, Germany) following the manufacturer’s instructions. The first strand cDNA synthesis was performed using RevertAid^™^ First Strand cDNA Synthesis Kit (Thermo Scientific, USA). Primers of the *CsGL3* were designed with Primer3web (http://primer3.ut.ee/). The primers for the *CsGL1* (*Mict*) gene were based on Zhao et al. [18]. The cucumber ubiquitin extension protein gene was used as the reference. Sequence information of all primers used in this study is provided in Additional file 1: Table S1. Quantitative real-time PCR (qPCR) was performed using the SYBR Green PCR master mix (Applied Biosystems Inc., USA) in the iCycler iQTM 5 Multicolor Real-Time PCR detection system (Bio-Rad, USA) following Xia et al. [47]. Relative quantification was calculated according to Livak and Schmittgen [48]. Each sample was run with three biological and technical replicates and significance tests among replications were performed with the *t*-test.

### Characterization of LTR_RT sequences and verification of insertion in natural populations

The mutation of *CsGL3* in RIL-46 M was due to the insertion of a 5005-bp LTR-RT. Self-alignment of the LTR-RT sequence was performed using CLC Genomics Workbench (V7.5) (http://www.clcbio.com/blog/clc-genomics-workbench-7-5/) with standard settings. The first and last 300 bp of this LTR-RT were aligned with each other to identify the LTR sequences with Clustal Omega (http://www.ebi.ac.uk/Tools/msa/clustalo/). Genes within the LTR-RT sequence were annotated with the FGENESH program, and conserved protein domains were predicted with BLASTp. The distribution of this LTR-RT sequence in the cucumber genome was examined. NGS sequencing reads of different cucumber lines were aligned with the BWA software using the 10,199 bp sequences of *csgl3* as the reference.

To investigate allelic diversity of the *csgl3* locus in natural populations, three primers, two (GL3_2L and GL3_2R) flanking the LTR-RT insertion points and one (INSERT_R) within the LTR-RT were designed for duplex PCR such that two PCR products of different sizes during agarose electrophoresis were expected in lines with and without the LTR-RT insertion. Primer sequences of the three markers are provided in Additional file 1: Table S1.

### Phylogenetic analysis of *CsGL3* candidate gene sequences

We investigated the phylogenetic relationships of cucumber CsGL3 protein with class IV HD-ZIP TF proteins identified in *Arabidopsis* [7], tomato (*Solanum lycopersicum*) [13], maize (*Zea mays*) [36, 49], rice (*Oryza sativa*) [16] and cotton (*Gossypium* spp.) [14, 15]. The cucumber *CsGL1* (class I HD-ZIP protein) amino acid sequence [17, 18] was included as an outlier. Multiple sequence alignment was performed with the Clustal Omega program (http://www.ebi.ac.uk/Tools/msa/clustalo/). The neighbor-joining tree [50] was constructed by the MEGA 5.0 software (http://www.megasoftware.net/) with 1000 bootstrap replications. The GenBank accession numbers of these 26 class IV HD-ZIP members were *PDF2* (NP_567274), *GL2* (NP_565223), *ATML1* (NP_193906), *ANL2* (NP_567183), *HDG1* (NP_191674), *HDG2* (NP_172015), *HDG3* (NP_180796), *HDG4* (NP_193506), *HDG5* (NP_199499), *HDG6* (NP_567722), *HDG7* (NP_200030), *HDG8* (NP_186976), *HDG9* (NP_197234), *HDG10* (NP_174724), *HDG11* (NP_177479) and *HDG12* (NP_564041) in *Arabidopsis*; *ZmOCL1* (CAG38614), *ZmOCL2* (CAB96422), *ZmOCL3* (CAB96423), and *ZmOCL4* (CAB96424) for maize; *GaHOX1* (ABY41242), *GaHOX2* (ABY67263), *GhHD1A* (AFO11041), and *GhHD1D* (AFO11042) for cotton; *OsRoc1* (BAB85750) and *OsRoc5* (BAC77158) for rice.

## Availability of supporting data

All the supporting data are included as additional files in the manuscript.

## Additional files

Additional file 1: Table S1. Primer information of all markers used in the study for genetic mapping, quantitative real-time PCR, allelic diversity study in natural populations. **Table S2.** Names, locations and predicted functions of 12 annotated genes in 68.4 kb region harboring the CsGL3 locus. (XLSX 16 kb) Additional file 2:
**Genomic DNA sequences for** ***CsGL3*** **in wild type** **RIL-46** **W (5188 bp) and** ***csgl3*** **of the glabrous mutant** **RIL-46** **M (10,199 bp).** Target insert point was marked with green color. LTR retrotransposon sequence insertion in the mutant is underlined. (DOCX 19 kb) Additional file 3: Figure S1. Detection of 5005-bp LTR retrotransposon insertion found at the *csgl3* allele in natural populations of cucumber. M: 1 kb markers; *csgl3*: the glabrous mutant RIL-46 M amplifies a 917 bp fragment suggesting presence of the insertion; 1 to 46: representative materials of the natural population (total 384) with 461 bp fragment showing no insertion of the LTR retrotransposon in these materials. (PPTX 138 kb)

## Abbreviations

BSA: Bulked segregant analysis
dCAPS: Derived cleaved amplified polymorphic sequences
ESEM: Environmental scanning electron microscope
EST: Expressed sequence tags
Indel: Insertion/deletions of DNA sequence
LTR-RT: Long terminal repeat type retrotransposon
NCBI: National Center for Biotechnology Information
qPCR: Quantitative real-time PCR
RIL: Recombinant inbred line
SNP: Single nucleotide polymorphism
SSR: Simple sequence repeat
TE: Transposable element
TSD: Target site duplication
WT: Wild type
