# Supplementary material for: The loss-of-function GLABROUS 3 mutation in cucumber is due to LTR-retrotransposon insertion in a class IV HD-ZIP transcription factor gene CsGL3 that is epistatic over CsGL1
Source: BMC Plant Biol. 2015 Dec 29;15:302. doi: 10.1186/s12870-015-0693-0 (PMC4696102; doi:10.1186/s12870-015-0693-0)
Supplement: Additional file 2: — Genomic DNA sequences for CsGL3 in wild type RIL-46 W (5188 bp) and csgl3 of the glabrous mutant RIL-46 M (10,199 bp). Target insert point was marked with green color. LTR retrotransposon sequence insertion in the mutant is underlined. (DOCX 19 kb) [file 12870_2015_693_MOESM2_ESM.docx]

**Supplemental file 1:** Genomic DNA sequences for *CsGL3* in wild type RIL-46W and *csgl3* of the glabrous mutant RIL-46M (10,199 bp). Target insert point was marked with green color. LTR retrotransposon sequence insertion in the mutant is underlined.

**>*CsGL3* (RIL-46W)**

**ATG**TTCGATCCAGATATGTTTGATACTACTCACCATCATATGCTTGAAGAGTTGGAAAATATGAGAGACGATGATTTTGACAACAAATCAGGTGCAGAAATCTTGGAATCTGCTTGTGGAACTGACCAACAACAACAACGTTCTAAAAAGAAACGTTACAATCGTCATACACAACATCAAATCCAAGAAATGGAAGCGTAAGTCGTCTGAACTACACCTATCCTTTTTTCCTCTTCTTCTTCATTTCAACGGAAAAAAATCAAAATAATTTTTTTGGTTTGATTTGTCAGATTCTTTAAGGAATGTCCTCACCCAGATGATAAGCAGAGAATGGAGCTGAGTCGTGAGCTGGGGTTGGAGCCATTGCAAGTCAAGTTTTGGTTTCAAAACAAACGCACACAAATGAAGGTGACCATACAAATCTATCTAATATTTTATTTTGGTGGAATAACATTTTTTTCTCTTTTCTTTTCCAAACTTTTGGATTTAGTTTCTAAAGTTTGAAAATGTTACACTTTTTTGTTAGTTCAGTTTCTAGGTTTTCAAATATTAACACTTTACATTTGAGATTTGTTTTGTTTCTAATTTAAAAGTTTACATTTTTTTGAATGATTTTTTCACTAAACACTCCTTTGTCATCTGAAATGTTAAGTGTGTATTAATTAAATTTCATTATTGTTCATTGCTATTAAAATATAGTGGAAAAACAAGTTTTAAAAAGTAACCTCCAAAATCTGTGACCTTATGACCCAAAACTTGGAAATAGGATTGTTTAACTGTGGTTGTGTTAATTTGAATGAGAGAAGGCTCAACACGAGAGACATGAAAACGCAATATTGAAGGCGGAAAATGAGAAACTTCGTGCTGAGAATATCCGCTATAGAGAAGCATTCGCTCATTCAACTTGCCCTAACTGCGGATCTTCCTCCACTGCACTCGGGGAAATGTCCTTTGACGACCAACATTTACGCATTGAAAATTCCCGCTTGCGAGATGAGGTCATATTCTTATTACCATTACTATTTCTCTTTTTTCTTTATGTCCATATATATATATATATATATATAGTGTGTGTGTGTGTATGTATGTATGTATGTATGTATCAAATACCTTTTTATATAACTTAAAATAGGTTATAAATAGCTGTACTGCAAGAAGTAAGCTAAAAAGTAACAATCCACCATATTCAGATTACTGTTCATTTTCTATATATATATATATATATATATATATATATATATATATATATATATATATATTTTGACATGGATTTCTCGTTTGGGTTGGTATGTAGTTCGTAGTTATTTTACGACTGAGGCCAGACATTCATCCTTGTAATTGTGACAGCAACTTATAAGATCTTTTTGGATAGATATGAGTAGAAAGAACTATAGCATTACAAGCAACCACATTCATTACTAAATTTATTATTCACAATAACATATACGGCGAGTTTATTGTTTGGCCCATTCCATTTTCATATTTTTCTCAACACATACCTCTCCAATCCCTATCATATTTACGTATTTATTTCCATTTCATCAAGTCTATTTTTGTCATTTTCCTCTATTTCCCTACTTTTTTTAAATATATATATATATATATGTATATATATATATATATATATAGTCAAACATTGCATTGTGTCTGTATAGGTGGGTGTAATGAGTGGTCACAATTCAAGTGATATTTCACATATTGTTCATCAATGAAGTAGGGAAATAGATATATTTATTTATTGAAGTTCTTAAGTTTTAGGTTGAGTTCTCATTTCTCTCTTGATTTCAAAAAATTATAAAGTATATTCCTGAAAGCTTCAATATTTACTTTTGCATTGAGAGAAAGAGAAATTAGGGTTAAAAACGTGGATATAAAATAGAAATAATTTAAAAAGAGAGAATCAAATAATAATTATATTAAAATTAAATGTAATAATAATTGGGTGTGAGTACTGAGGAGTACTGCAGTGGGTGTGGGGAGAGATCATAATTACCCGAAATTAGGAAATAGAGATTTTTATGATTCTTGTGAATTTATAACACTGTGTCCTTTTCCGCCTCTTGTCATAAATTTTTAATTCAATTTTTAGTCCATTAAATATTTTCGTACTTTCCCTTTTGCTTTCATCCACAAAAAAAAAAAAAAAAAAACACATTAAATTTTGTTTACACTCTGAATTTATTTTTGTAAGCGCTATCAATTACACATCATTACAAAAATATAAGACCTTTTCTCCAACTCTTTAACTTCAAATATAAATTGCTTTTGTAATGTTTTGATTATTATACATTCAAGTATGACATTTACTTTATTTATAATTCGTTAACAAACAAACATCTTCAAAACAACTATCTTCTTCCATTTTAGATTTAAATCGATTTTTCTTAATACCAAATACCATTATCCTAAGAAAAAGTGATTTCGCAAAGAAAATAGAATTTAAATCACTTCAAAACAATCCCTAAATTGGTTGGATCAAAATCCCGAATTCTAAAATCATCAACAAAGATGCTGTAGTTTTTGTTTGGAAGTGTTTCAATTTATATGCTGATTTTGAGGTCTCATTGATGATTATACACGAGACAGATCGAGCGAATGAGTGGATATGGCTCAAAATGCACAAAGCCATACTATCAGCTTCCAACAAATGCTCCAACGCGATCTTTAGACCTGGGCATCACTAACTTCGGTCCACAGTCGTCAGGATTCGTAGGGGAAATGTATGGAGCAGCAGATTTTTTCCGGTCGATATCGAGGCCTTCGGAAGGTGAGAAACCGGTGATCGTGGAGCTGGCGGTATCGGGGATGGAGGAGTTGAGGAGAATGGCTCAGGGAGGGGAGCCGTTGTGGGTTGCGGGAGATGGGAAGTCATCAGGGGAAGTGGTTCTTAATGAGGCGGAGTATCTGAGGAGTTTCGGAGGGGGAATAGTGGGAAAGCCGATGGGCTTTAGAACCGAAGCTTCACGAGTTTCGGCTGTGGTGTTCATGAACCATATGAAGTTGGTGGATATTTTTATGGATGCTGTACGTTTCATTCAAGATTTTCTCTCTGAATTACTATTAGTTATGTAAATTATTACTTATTAATAACAATGTATTTGTTTGTTGTTTTTAGACGCAATGGTCCACTGTGTTTTGTGGCATTGTTTCGAGAGCCTCCACTGTGGAAATTCTTTCGCCTGGCTTACCTGGAAACTTCAATGGGGCCTTGCATGTGGTACTTAAACTAAACCCTCTCTCACTCCTTTACTTTTGGTGATTTTGTTCTTGACTATGCAAAATTTGGTACATAATAAATATAAAAATTTCACAAAAAGATTAATTAATCATTACAAATTTAAAAACAATTAAAGGGAACTAATTCAATAATTCATCACACGTTAAACTTCAAAAGACTAAATAATTATAACCAATATAAAGTATAAAAATTGAGTTTTGTTTCATAACCATTTAACTTTTAAAATTATTATTTGTTATTTTCAATATGTATATAATGACATTATTTTTCACTTGTGAGATTTTTATATAGACATTTGAATTCAGATAGCTAATTTTTTTTAAAATCCTTTGAAAACAAAATTGACTTTGATTTGGTTGATTTTGAAAAGAAATCTAACGTTTAGACAACGATGTATATGAATAAAATAAAAATATAATGGAAAGTTTGGTATAAAAATTGATGGGTTCTGTATTAAACGAACAGATGAGCGCTGAATTTCAAGTCCCATCGCCACTTGTTCCAACTCGTGAAAACTATTTCGTAAGATACTGCAAACAACAAACTGATGGTTCATGGGCTGTGGCTGATGTTTCCTTAGACACCTTGCGCCCTTCTCCCATCCCAAATACCCGAAGGAAACCCTCTGGTTGCTTAATTCAAGAATTACCCAACGGCTATTCCAAGGTTACAGAACGCTCATTCTCTCTTTAACAACCCTTTCATTCAGCATCATCTCATTTCTGATCTTTATTTAACTTTGGATCATTTATTCCTTCTTCAGATCACCTGGGTCGAACATGTAGAAGTCGACGAAACTGGCGTCCCCACAATGTACAGGACTCTTGTTAATTCCGGCCTCGCTTTCGGGGCTAAGAGATGGGTTGCTACCTTAGATCGCCAATCTGAGCGTTTCGCTACTTCAATCGCCACCACCATTCCCACCGGCGATTTACGCGGTATTACTCTACTTTCTTCATTCTTCTGTTTCCCCCGGTTTTTCCTCATCATTCCCACTAATTTGATTTAATTTAAATTCAGTGATCTCAAGTATTGAAGGGAGGAAGAGTATGCTGAAACTAGCAGAGAGAATGGTGACGAGCTTCTGCGCCGGTGTAGGTGCGTCCAGTGTACACGCTTGGACGGCATTACCCGCTGCAGCTGGCGATGAAGTACGTGTTGTGACGAGGAAGAGCACTGACGAACCAGGACGACCACCAGGTGTTGTGTTGAGCGCCGCCACTTCTTTTTGGATTCCAGTTTCACCTAAGGTTGTTTTTGATTTTCTTCGAAAAGAGAAATCTCGGAGCGAGGTACGTATGTAGCATATATATAAACATCAAAACATTGAAACAATCGTTAAGTAAACAGAGTATTGAAATATTTGCAATATTTTTTCAGTGGGATATTCTGTCAAATGGGGGATTGGTTCAGGAAATGGCGCACATAGCCAACGGCCGTCACTCAGGGAATTGTGTCTCCTTGCTTCGTGTTAATGTCAGTACATTATTCTCTTCTTTTTTAAAAAAAATTGAGGAACAGTTATTAGTTAACATATAAATCAATGAAAATTCTAAACTTGGTTATCTTTTGTAGAGTGCGAATTCGAGCCAGAGCAATATGCTGATACTACAAGAAAGCTGCACGGACTCAACAGGATCGTACGTTATTTATGCTCCGGTGGACACCGTGGCAATGAACGTGGTGCTAAGTGGTTGCGATCCGGATTACGTGGCGCTTCTACCGTCGGGATTCGCCATACTTCCTGACGGACCCGGCGGAGGAGGAAACAACGGCGGCGGCATTCTAGAGCTGGGATCGGGTGGGTCACTCATCACGGTTGCATTTCAAATCCTGGTTGATTCGGTTCCAACGGCTAGACTTTCAATTGGATCAGTAGCCACCGTTAACAGTCTCATCAAATGCACGGTTGAGAGAATCAGAGCCGCTGTGATGCGTGAAAACCCA**TGA**

**>*csgl3* (RIL-46M)**

**ATG**TTCGATCCAGATATGTTTGATACTACTCACCATCATATGCTTGAAGAGTTGGAAAATATGAGAGACGATGATTTTGACAACAAATCAGGTGCAGAAATCTTGGAATCTGCTTGTGGAACTGACCAACAACAACAACGTTCTAAAAAGAAACGTTACAATCGTCATACACAACATCAAATCCAAGAAATGGAAGCGTAAGTCGTCTGAACTACACCTATCCTTTTTTCCTCTTCTTCTTCATTTCAACGGAAAAAAATCAAAATAATTTTTTTGGTTTGATTTGTCAGATTCTTTAAGGAATGTCCTCACCCAGATGATAAGCAGAGAATGGAGCTGAGTCGTGAGCTGGGGTTGGAGCCATTGCAAGTCAAGTTTTGGTTTCAAAACAAACGCACACAAATGAAGGTGACCATACAAATCTATCTAATATTTTATTTTGGTGGAATAACATTTTTTTCTCTTTTCTTTTCCAAACTTTTGGATTTAGTTTCTAAAGTTTGAAAATGTTACACTTTTTTGTTAGTTCAGTTTCTAGGTTTTCAAATATTAACACTTTACATTTGAGATTTGTTTTGTTTCTAATTTAAAAGTTTACATTTTTTTGAATGATTTTTTCACTAAACACTCCTTTGTCATCTGAAATGTTAAGTGTGTATTAATTAAATTTCATTATTGTTCATTGCTATTAAAATATAGTGGAAAAACAAGTTTTAAAAAGTAACCTCCAAAATCTGTGACCTTATGACCCAAAACTTGGAAATAGGATTGTTTAACTGTGGTTGTGTTAATTTGAATGAGAGAAGGCTCAACACGAGAGACATGAAAACGCAATATTGAAGGCGGAAAATGAGAAACTTCGTGCTGAGAATATCCGCTATAGAGAAGCATTCGCTCATTCAACTTGCCCTAACTGCGGATCTTCCTCCACTGCACTCGGGGAAATGTCCTTTGACGACCAACATTTACGCATTGAAAATTCCCGCTTGCGAGATGAGGTCATATTCTTATTACCATTACTATTTCTCTTTTTTCTTTATGTCCATATATATATATATATATATATAGTGTGTGTGTGTGTATGTATGTATGTATGTATGTATCAAATACCTTTTTATATAACTTAAAATAGGTTATAAATAGCTGTACTGCAAGAAGTAAGCTAAAAAGTAACAATCCACCATATTCAGATTACTGTTCATTTTCTATATATATATATATATATATATATATATATATATATATATATATATATATATTTTGACATGGATTTCTCGTTTGGGTTGGTATGTAGTTCGTAGTTATTTTACGACTGAGGCCAGACATTCATCCTTGTAATTGTGACAGCAACTTATAAGATCTTTTTGGATAGATATGAGTAGAAAGAACTATAGCATTACAAGCAACCACATTCATTACTAAATTTATTATTCACAATAACATATACGGCGAGTTTATTGTTTGGCCCATTCCATTTTCATATTTTTCTCAACACATACCTCTCCAATCCCTATCATATTTACGTATTTATTTCCATTTCATCAAGTCTATTTTTGTCATTTTCCTCTATTTCCCTACTTTTTTTAAATATATATATATATATATGTATATATATATATATATATATAGTCAAACATTGCATTGTGTCTGTATAGGTGGGTGTAATGAGTGGTCACAATTCAAGTGATATTTCACATATTGTTCATCAATGAAGTAGGGAAATAGATATATTTATTTATTGAAGTTCTTAAGTTTTAGGTTGAGTTCTCATTTCTCTCTTGATTTCAAAAAATTATAAAGTATATTCCTGAAAGCTTCAATATTTACTTTTGCATTGAGAGAAAGAGAAATTAGGGTTAAAAACGTGGATATAAAATAGAAATAATTTAAAAAGAGAGAATCAAATAATAATTATATTAAAATTAAATGTAATAATAATTGGGTGTGAGTACTGAGGAGTACTGCAGTGGGTGTGGGGAGAGATCATAATTACCCGAAATTAGGAAATAGAGATTTTTATGATTCTTGTGAATTTATAACACTGTGTCCTTTTCCGCCTCTTGTCATAAATTTTTAATTCAATTTTTAGTCCATTAAATATTTTCGTACTTTCCCTTTTGCTTTCATCCACAAAAAAAAAAAAAAAAAAACACATTAAATTTTGTTTACACTCTGAATTTATTTTTGTAAGCGCTATCAATTACACATCATTACAAAAATATAAGACCTTTTCTCCAACTCTTTAACTTCAAATATAAATTGCTTTTGTAATGTTTTGATTATTATACATTCAAGTATGACATTTACTTTATTTATAATTCGTTAACAAACAAACATCTTCAAAACAACTATCTTCTTCCATTTTAGATTTAAATCGATTTTTCTTAATACCAAATACCATTATCCTAAGAAAAAGTGATTTCGCAAAGAAAATAGAATTTAAATCACTTCAAAACAATCCCTAAATTGGTTGGATCAAAATCCCGAATTCTAAAATCATCAACAAAGATGCTGTAGTTTTTGTTTGGAAGTGTTTCAATTTATATGCTGATTTTGAGGTCTCATTGATGATTATACACGAGACAGATCGAGCGAATGAGTGGATATGGCTCAAAATGCACAAAGCCATACTATCAGCTTCCAACAAATGCTCCAACGCGATCTTTAGACCTGGGCATCACTAACTTCGGTCCACAGTCGTCAGGATTCGTAGGGGAAATGTATGGAGCAGCAGATTTTTTCCGGTCGATATCGAGGCCTTCGGAAGGTGAGAAACCGGTGATCGTGGAGCTGGCGGTATCGGGGATGGAGGAGTTGAGGAGAATGGCTCAGGGAGGGGAGCCGTTGTGGGTTGCGGGAGATGGGAAGTCATCAGGGGAAGTGGTTCTTAATGAGGCGGAGTATCTGAGGAGTTTCGGAGGGGGAATAGTGGGAAAGCCGATGGGCTTTAGAACCGAAGCTTCACGAGTTTCGGCTGTGGTGTTCATGAACCATGTAAACAATATGGAGAATCTTCTTTTATTTTGTTATATAAAATCAAGAATACAATGATTTATATACAGTTAATTTGGACAACCTATAAGGAAAGGATTAAATTAATCTAATTACAATAATGATTCCTAAATTACAGCTAAATTACTTTAATTACGGTGATAATTAATCTAATTACAATAATGATTCCTAAATTACAGCTAAATTACTTTAATTACGGTGATACCCTCCCTCAAACTCAAGTTGGTAGAGTAGAAACCACCTTGAGTTTGTGAAGTAACAGAGTGAAACGAGGAGAATGGAGAGCTTGGGTGAAGATATCTGCAGGTTGATCAATGGTAGAGATAGATTGAAGATGGAGAGTGTTGCTTTGAAGATGATGACGAACAAAATGACAATCGTTCTCTATGTGCTTTGTTCGTTCATGAAATACATCATTGTGTGCAATCTGAATGGCACTGCGGTTATCACAATGGAGAGTAGTAGATGATGTTTGTGGGGCTCCCATATCAGTAAGAAGCCAACGAAGCCATAATAATTCTGAAGTGGCATCAGCTAGAGCACGATATTCAGACTCAGTGCTAGACCGGGAAACAACAGATTGTTTCTTGCTGCGCCAGGAGATAAGAGCATCACCTAAATAAAAACAATAGCCAGTGGTGGATCTTCTATCAGTAGGATCTCCTGCCCAATCAGCATCAGAGAATCCAGAGAGAACCAGAGAAGACTGTGAGGAGAAGTGGAGACCATGACCCAAAGTGCCTTTAATGTAGCGAAGAATCCGAAGGACAGCAGTGAAATGAATAGTACGGGGAGCGGCCATGAATTGACTGACTATGTGAACTGCGAATGCAATATCAGGGCGAGTTACAGTTAAGTAAATCAAGCTACCAACAAGTTGCCGGTACAAAGTAGGATCGTCAAGGGGGACACCATCATAAGGAGTAAGTCGAACATTTGGATCCAGAGGTGTTGAGGATGTTGTAGAATCAGTAATACCAGATCGACTGAGTAGGTCAGAAGCATATTTAGCTTGAGATAAGTAATAGCCATCAGATACGGAGGTGATCTCAAGGCCAAGGAAATAACTGAGAGGTCCTAGATCCTTCATTTCAAAGTGCTTTCCCAGGTAGCATTGCAATTCAGATATAGCTTGAGGGTCATCGCCTGTAATAATCATATCATCAACATATAAAAGAAAGGAGTACAATACCATTATGTGTCTGGCGGGTGAACAAGCTGAATCATGAGGGCTGGAAGTGAACCCAAGTTGAGTAATAGTGGAGCTAAAAGTTGCAAACCAGCTCGGGGAGCCTGTTTGAGACCATAGAGAGCTCGTCGAAGAAGACATACTTTCTGATGTGGCGGGGTAGTACCAGGTGGTGGTTTCATATAGACTTCTTCAGATAGAGTTCCATTGAGAAATGCATTTTTAACATCCATTTGTAAGAGGGGCCACTGTTTTGCAGCTGCAATGGCTAAAAGACTACGAACAGACGTCATTCGAGCTACTGGAGCAAACGTTTCTTCATAATCAATGCCATATTCTTGAGAATAACCTTTGGCTACAAGTCGTGCTTTGTATCGTTCTATAGACCCATCAGAGTGCGTTTTGATTTTGAAGATCCATTTACAACCAATAGGTTTCTTTCCAGGAGGTAAGTCCACATATTCCCAAGTATGAGTCTTTTCTAAGGCTTGAAGTTCTTCATTCATTGCTTGCTGCCATAATGGGTTAGTGCTGGCTTCCTTATACGAGGAAGGTTCAACTAAAGACATGATGGTGGAGAAACAGTGATAATCTTTAAGATGAGAGGGAATTTCTCTTACCCGTGTAGATCGTCGGATTGGTGTAGATTCAGGTTCCTCAAGAGGGGCAGATGGAAGATCCGGGAGTGCAGAAATATGGTCCGATTGAGTGGGCTCAGGTATAGGTAACATGCAAGGTGTAGTGTCCGGTGTTGAATCAAGTGAGGGGAAAAAGATCAATAGACGAATCGGTGAAAAATGGGTGAGAACTTGAAAGAGAATTCTGGGAATGAGGAAGGACTAAAAACATACGATGTTCCCAGAAGGTGACATGACGAGATATACGTAATCGTTGAGAGATGGGATCCCAACAACGAAATCCTTTATGTTCAGTACCATAACCCAAGAAGCAACATAGACGTGCACGTGGTTCAAGTTTGGTATGTTCATGAGGATATAATAATACGAAACATGCACAACCAAAAATTTTTAGATTGGAGTAAGAAGGGGAAGTACCGTATAGTCTTTCAAAGGGAGAAACATTTTTAGTGACTTTTGAAGGAAGACGGTTGATGACATAGACAGAGGTGAGGGCAGCCTCGCCCCAAAATTTTTCTGGACAAGCAGCAGATAGAAGCTGAGCACGAACAGAGTCAAGAATGTGACGATGTTTGCGTTCAGCTCTTCCATTTTGCTGAGAGGTGTGAGGACAGGAGCGTTGAATCAGAGTGCCTTGCTGGGCAAGGAAGGAAAGAAGTTGAGAGTCCTTATATTCCATCGCATTATCAGTTCGAAAGATTTTAATTTTGCTGGAGAATTGAGTGTGAATCATATTTGCAAAATCAACATATATTTGATATAAGGAAGAACGATTTTTAAGAAAGTAAATCCAAGTAAAACGAGAATAGTCATCAATAAATAAGACAAAGTATCGATAACCATTAATGGTAGTACAAGGAGCAGGACCCCAAATGTCAGAATGAATAAGGTCAAAAGGCTTATTACATAGAGAGGTGGAATTAGGAAATGATAAAGCAGGTTGTTTTGCAAGTTTGCAATTTAAGCAATCAAAATGACTGAATTTTGAGACATTATTCAAATTACCAGTAGAAACTAAATTACGAAGTTTATCAGAGGATGCATGACCTAGACGAAGGTGCCACTGATATATAGAGGTATCAGTGACTGGTGCAGAGATAGGTGGAAACTCAGGAGATTGCTGAAGATATGTAAGCTCAAATAATCGTCCCACCTTCCGTCCCGTACCAATCACTTGCCCCGTTTGAGAATCCTGAACCTGACACCCATGAGAAGAAAAGATAACAGTTAGTCCGAGATCACAAAGTTGGCCAACAGAAGCTAGGTTGAATGTGAGTTTTGGAACATGATAGGTGTTTGACAGTTTTATTGTGGGTGTGTTAACAGTACCAACGTGAGAGATAGGCATATGATTACCATCAGCAGAGTGAATTGAGGGAAGAGATTGAACATGAGTAGGTGAGGATAATAATGAAATGTCAGCAGTCATATGGTTGCAACAAGCTGAATCAAGGAGCCAAGATGTACCTGGAGTGACGGCAAGAGCAGTAGAGTTGGAAGAGATCACCTGTTTCAGTAAGTCATGAAGATCATTTAACTGAAAATTCTGAGGTGTGGTGATATCAGATGGGGTAGCAGCAGCAACAATAGGTGGAGAACTGTATTTAGTGGAAGACTTGGGTTTATATGGAGCACCAGGAGGACGGGGGTGGACGGGTGGGACAGTTGTCCAAGATATGACCTCGTTTGTGGCAATACCTGCACTCAATAGTAGGACAATTAGCAAACTTATGACCATGAAGTTTGCAATTTTTGCAGAAAATAGACTCATTAGCCGATCGTGAATGGGTGGTTGCAAGAGCGACATCAGATGGCAGAGAGGAAACAATGCCAAGGCGGTTTTCCTCAAATAAGATATCTTGTATGGCAGCATCAAGACTCGGCAGAGGGTTACGATGTAACAATGCGGCACGGACTGATTCATACTCTGGGCGAAGACCCATAAGAACTTTGATGAGGCGAATATGCTCCGGATTGATTTTTGCTTGATCTAACTGAGTCCATATTGGTTGAAGAGTTGCAAGATACTCGTTCACAGATTGTCCTCCCTCCTGATTTAGACTAACAAGTCGAGAGTGCAATTGGTAATAGTGAGCAAGTCCAATAGACTGAAAACGTGTGGACAGAAAATCCCAGAGTTCTTTTGCCGTATCAAATGCATCAAATTGTGTGTGAATGGCTGGAATAGAGGTGTTACCTAACCAAGTAATGATCTGATGGTTTTTGCTATCCCAATCCTCAAGACGTTCAATGTATTTGTCGGTTTCTGCAGTCGTCTCTTGAACAAGTGACATCAGATGGGTCTGTATCAATTATTTTCAGGTGGTGTCTATCGAAGGAGTAGGTTTGACTGGTTAGTGATATCTCCAGTAACGATGCGCCACAATTTCGACCTATCAAAAAACTCCCTCATTGGTGGGCCCAAGTGATATATTTAGTTCCATCGAGAATGGTGCTAATGGGACGAGCAACTTTCATGTTTCTCCATCGAAAACGGCAACTCTTCAACTCTCGGACAAAAACAAGACGGCTGGTGTGAGTGGCAGACGCGCGCGAGATCTGAAATCGTGGACAGAACTAGAACCACTGATAGATCTGATTCAACGTGTGTGGTTTGCTTCAGAGATCTCTTCGGGCGGCGACTAGGAAGATTGATGGTCAGATGGTTCGGCGAAAAATGGCGGACGTGGGTGACAAGCAGAGCGAGGATTGGCAGGTGGCGTGAGCGACTGACGCGTGTGAGATCTGGGATCGTGGACAGAAGAAGAGTCACAGACAGATCTGGTGTGAGTGACAGATGTGTACGACTTGCTTCAGAGATCTCGTCGGAGATCTGAACGGAGGTGGTCGTCGGAGAGTTGAACGGGAGATGACCGGCGGACGGAATTGAACTAGCAGCGGCGAAATTTCTAAAAGAAAATTAGGGCTCTGATACCATGTAAACAATATGGAGAATCTTCTTTTATTTTGTTATATAAAATCAAGAATACAATGATTTATATACAGTTAATTTGGACAACCTATAAGGAAAGGATTAAATTAATCTAATTACAATAATGATTCCTAAATTACAGCTAAATTACTTTAATTACGGTGATAATTAATCTAATTACAATAATGATTCCTAAATTACAGCTAAATTACTTTAATTACGGTGATAAACCATATGAAGTTGGTGGATATTTTTATGGATGCTGTACGTTTCATTCAAGATTTTCTCTCTGAATTACTATTAGTTATGTAAATTATTACTTATTAATAACAATGTATTTGTTTGTTGTTTTTAGACGCAATGGTCCACTGTGTTTTGTGGCATTGTTTCGAGAGCCTCCACTGTGGAAATTCTTTCGCCTGGCTTACCTGGAAACTTCAATGGGGCCTTGCATGTGGTACTTAAACTAAACCCTCTCTCACTCCTTTACTTTTGGTGATTTTGTTCTTGACTATGCAAAATTTGGTACATAATAAATATAAAAATTTCACAAAAAGATTAATTAATCATTACAAATTTAAAAACAATTAAAGGGAACTAATTCAATAATTCATCACACGTTAAACTTCAAAAGACTAAATAATTATAACCAATATAAAGTATAAAAATTGAGTTTTGTTTCATAACCATTTAACTTTTAAAATTATTATTTGTTATTTTCAATATGTATATAATGACATTATTTTTCACTTGTGAGATTTTTATATAGACATTTGAATTCAGATAGCTAATTTTTTTTAAAATCCTTTGAAAACAAAATTGACTTTGATTTGGTTGATTTTGAAAAGAAATCTAACGTTTAGACAACGATGTATATGAATAAAATAAAAATATAATGGAAAGTTTGGTATAAAAATTGATGGGTTCTGTATTAAACGAACAGATGAGCGCTGAATTTCAAGTCCCATCGCCACTTGTTCCAACTCGTGAAAACTATTTCGTAAGATACTGCAAACAACAAACTGATGGTTCATGGGCTGTGGCTGATGTTTCCTTAGACACCTTGCGCCCTTCTCCCATCCCAAATACCCGAAGGAAACCCTCTGGTTGCTTAATTCAAGAATTACCCAACGGCTATTCCAAGGTTACAGAACGCTCATTCTCTCTTTAACAACCCTTTCATTCAGCATCATCTCATTTCTGATCTTTATTTAACTTTGGATCATTTATTCCTTCTTCAGATCACCTGGGTCGAACATGTAGAAGTCGACGAAACTGGCGTCCCCACAATGTACAGGACTCTTGTTAATTCCGGCCTCGCTTTCGGGGCTAAGAGATGGGTTGCTACCTTAGATCGCCAATCTGAGCGTTTCGCTACTTCAATCGCCACCACCATTCCCACCGGCGATTTACGCGGTATTACTCTACTTTCTTCATTCTTCTGTTTCCCCCGGTTTTTCCTCATCATTCCCACTAATTTGATTTAATTTAAATTCAGTGATCTCAAGTATTGAAGGGAGGAAGAGTATGCTGAAACTAGCAGAGAGAATGGTGACGAGCTTCTGCGCCGGTGTAGGTGCGTCCAGTGTACACGCTTGGACGGCATTACCCGCTGCAGCTGGCGATGAAGTACGTGTTGTGACGAGGAAGAGCACTGACGAACCAGGACGACCACCAGGTGTTGTGTTGAGCGCCGCCACTTCTTTTTGGATTCCAGTTTCACCTAAGGTTGTTTTTGATTTTCTTCGAAAAGAGAAATCTCGGAGCGAGGTACGTATGTAGCATATATATAAACATCAAAACATTGAAACAATCGTTAAGTAAACAGAGTATTGAAATATTTGCAATATTTTTTCAGTGGGATATTCTGTCAAATGGGGGATTGGTTCAGGAAATGGCGCACATAGCCAACGGCCGTCACTCAGGGAATTGTGTCTCCTTGCTTCGTGTTAATGTCAGTACATTATTCTCTTCTTTTTTAAAAAAAATTGAGGAACAGTTATTAGTTAACATATAAATCAATGAAAATTCTAAACTTGGTTATCTTTTGTAGAGTGCGAATTCGAGCCAGAGCAATATGCTGATACTACAAGAAAGCTGCACGGACTCAACAGGATCGTACGTTATTTATGCTCCGGTGGACACCGTGGCAATGAACGTGGTGCTAAGTGGTTGCGATCCGGATTACGTGGCGCTTCTACCGTCGGGATTCGCCATACTTCCTGACGGACCCGGCGGAGGAGGAAACAACGGCGGCGGCATTCTAGAGCTGGGATCGGGTGGGTCACTCATCACGGTTGCATTTCAAATCCTGGTTGATTCGGTTCCAACGGCTAGACTTTCAATTGGATCAGTAGCCACCGTTAACAGTCTCATCAAATGCACGGTTGAGAGAATCAGAGCCGCTGTGATGCGTGAAAACCCA**TGA**
