# Supplementary figures and images for: The loss-of-function GLABROUS 3 mutation in cucumber is due to LTR-retrotransposon insertion in a class IV HD-ZIP transcription factor gene CsGL3 that is epistatic over CsGL1
Source: BMC Plant Biol. 2015 Dec 29;15:302. doi: 10.1186/s12870-015-0693-0 (PMC4696102; doi:10.1186/s12870-015-0693-0)

## Slide 1
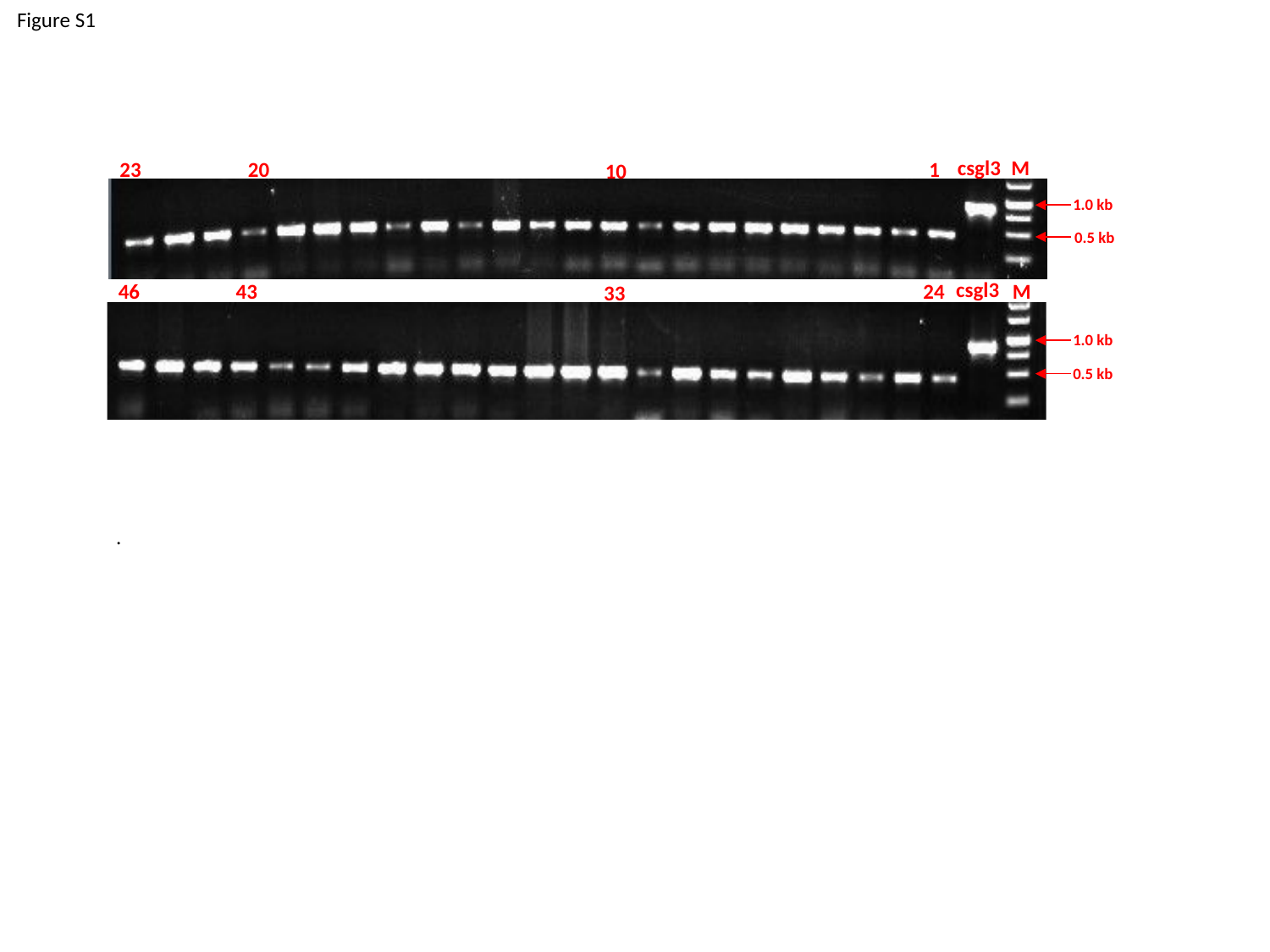

Figure S1
M
csgl3
23
20
1
10
1.0 kb
0.5 kb
csgl3
46
43
24
M
33
1.0 kb
0.5 kb
.

Supplement: Additional file 3: Figure S1. — Detection of 5005-bp LTR retrotransposon insertion found at the csgl3 allele in natural populations of cucumber. M: 1 kb markers; csgl3: the glabrous mutant RIL-46 M amplifies a 917 bp fragment suggesting presence of the insertion; 1 to 46: representative materials of the natural population (total 384) with 461 bp fragment showing no insertion of the LTR retrotransposon in these materials. (PPTX 138 kb) [file 12870_2015_693_MOESM3_ESM.pptx]
